# A Large Endoplasmic Reticulum-Resident Pool of TRPM1 in Retinal ON-Bipolar Cells

**DOI:** 10.1523/ENEURO.0143-18.2018

**Published:** 2018-07-04

**Authors:** Melina A. Agosto, Ivan A. Anastassov, Michael A. Robichaux, Theodore G. Wensel

**Affiliations:** 1 Verna and Marrs McLean Department of Biochemistry and Molecular Biology, Baylor College of Medicine, Houston, TX 77030

**Keywords:** bipolar cell, protein trafficking, secretory pathway, TRPM1

## Abstract

The chemical signal of light onset, a decrease in glutamate release from rod and cone photoreceptors, is processed by a postsynaptic G protein signaling cascade in ON-bipolar cells (BPCs). The metabotropic glutamate receptor mGluR6, along with other cascade elements, is localized synaptically at the BPC dendritic tips. The effector ion channel protein transient receptor potential melastatin-1 (TRPM1), in contrast, is located not only at the dendritic tips but also in BPC bodies and axons. Little is known about the intracellular localization of TRPM1, or its trafficking route to the dendritic tip plasma membrane. Recombinant TRPM1 expressed in mammalian cells colocalized with endoplasmic reticulum (ER) markers, with little or none detected at the plasma membrane. In mouse retina, somatic TRPM1 was similarly intracellular, and not at the plasma membrane. Labeling of ER membranes by expression of a fluorescent marker showed that in BPCs the ER extends into axons and dendrites, but not dendritic tips. In cell bodies, TRPM1 colocalized with the ER, and not with the Golgi apparatus. Fluorescence protease protection (FPP) assays with TRPM1-GFP fusions in heterologous cells revealed that the N and C termini are both accessible to the cytoplasm, consistent with the transmembrane domain topology of related TRP channels. These results indicate that the majority of TRPM1 is present in the ER, from which it can potentially be transported to the dendritic tips as needed for ON light responses. The excess of ER-resident TRPM1 relative to the amount needed at the dendritic tips suggests a potential new function for TRPM1 in the ER.

## Significance Statement

Retinal bipolar cells (BPCs) of the ON subtype detect signals from both rod and cone photoreceptors and are required for dim light vision. Little is known about the secretory pathway in these cells, or the trafficking of synaptic signal cascade proteins to their destination at the dendritic tip plasma membrane. The ion channel protein transient receptor potential melastatin-1 (TRPM1) is required for the depolarizing light response of ON-BPCs. However, in addition to its postsynaptic location at the BPC dendritic tips, TRPM1 is also present in cell bodies and axons. Our results show that somatic TRPM1 is intracellular and located in the endoplasmic reticulum (ER), and not at the plasma membrane, suggesting that plasma membrane insertion occurs specifically at the dendrites or dendritic tips.

## Introduction

At the synapses between photoreceptors and downstream bipolar cells (BPCs) in the retina, signals are divided into two channels: the ON channel, which responds to light onset, and the OFF channel, which responds to dark onset. Rod BPCs are all in the ON channel. In rod and cone ON-BPCs, glutamate released from photoreceptor terminals is detected by a metabotropic glutamate receptor signaling cascade. The glutamate receptor, mGluR6, signals via the heterotrimeric G protein G_o_, which in turn gates a transduction channel by an unknown mechanism (for review, see Martemyanov and Sampath, 2017). Transient receptor potential melastatin-1 (TRPM1) is required for channel function (Audo et al., 2009; Li et al., 2009; Morgans et al., 2009; Shen et al., 2009; Van Genderen et al., 2009; Koike et al., 2010b), but it is not known if it is sufficient for formation of the G_o_-sensitive channel (Lambert et al., 2011; Agosto et al., 2014; Schneider et al., 2015; Martemyanov and Sampath, 2017). In the dark, tonic release of glutamate activates mGluR6, keeping the channel closed; at light onset, decrease in glutamate release and deactivation of mGluR6 lead to channel opening and membrane depolarization.

**Figure 1.**
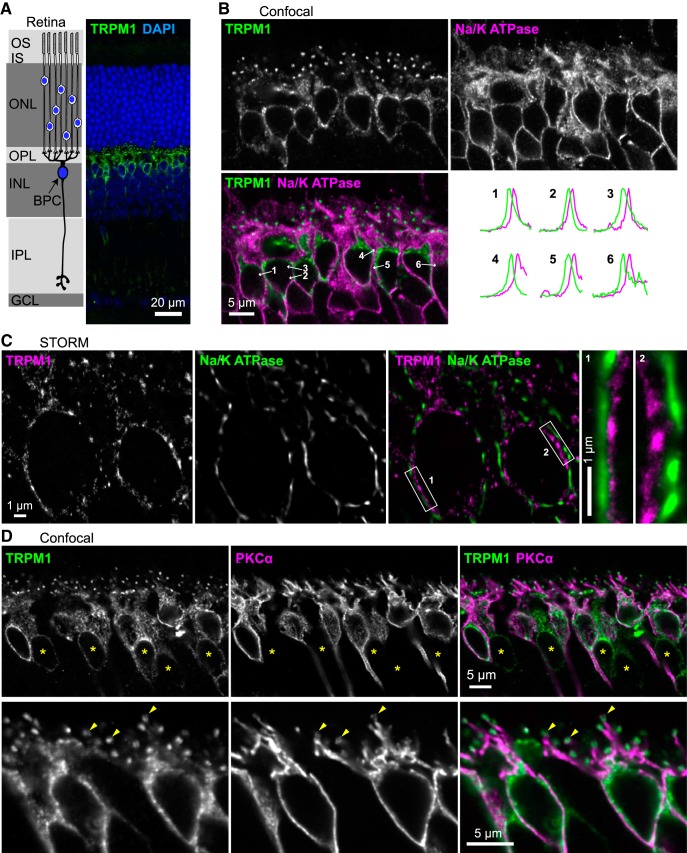
TRPM1 is intracellular in ON-BPC bodies. ***A***, Diagram of rod photoreceptors synapsing onto a rod BPC, alongside TRPM1 immunostaining (green) of a mouse retina section. OS, outer segments; IS, inner segments; ONL, outer nuclear layer; OPL, outer plexiform layer; INL, inner nuclear layer; IPL, inner plexiform layer; GCL, ganglion cell layer. ***B***, Retina sections were immunostained with antibodies for TRPM1 (green) and the plasma membrane marker Na^+^/K^+^ ATPase (magenta). Intensity profiles were measured along lines indicated by the arrows on the merged image and are shown normalized to the maximum height. ***C***, STORM reconstructions of retina sections immunostained for TRPM1 (magenta) and Na^+^/K^+^ ATPase (green). Boxes show the regions magnified on the right. ***D***, Retina sections immunostained for TRPM1 (green) and cytoplasmic protein PKCα (magenta). TRPM1 staining is also present in PKCα-negative cone BPC bodies (asterisks). Magnified images are shown on the bottom, with arrowheads indicating dendritic tips.

Retinal BPC bodies reside in the distal part of the inner nuclear layer; dendrites extend into the outer plexiform layer, where the dendritic tips form synapses with photoreceptor terminals, and the axon extends into the inner plexiform layer, where it forms synapses with amacrine or ganglion cells (for review, see Dunn and Wong, 2014; Fig. 1*A*). Like other secondary neurons, retinal BPCs are highly polarized and maintain specialized postsynaptic machinery specifically in dendritic tips. Disruption or knock-out of other synaptic components, namely, mGluR6 (Cao et al., 2011; Xu et al., 2012; Gregg et al., 2014), nyctalopin (NYX; Pearring et al., 2011; Gregg et al., 2014), LRIT3 (Neuillé et al., 2015), or ELFN1 (Cao et al., 2015), reduces or eliminates TRPM1 accumulation at the dendritic tips. However, localization of mGluR6 (Morgans et al., 2009; Koike et al., 2010b; Peachey et al., 2012a) and NYX (Pearring et al., 2011; Gregg et al., 2014) appear normal in TRPM1 knock-out mice, suggesting that these proteins do not require interaction with TRPM1 for trafficking. Consistent with this, a recent study of TRPM1 and mGluR6 during postnatal development found that mGluR6 appears at the dendritic tips earlier than TRPM1, and that these proteins are likely trafficked independently of each other (Anastassov et al., 2017). Despite these findings, protein trafficking in the BPC dendrites is still poorly understood, and little is known about the location of secretory pathway organelles in these cells.

Although the transduction channel activity of TRPM1 is likely restricted to the synapses at BPC dendritic tips, where other participants of the pathway are localized (Nomura et al., 1994; Masu et al., 1995; Morgans et al., 2006, 2007; Peachey et al., 2012b; Orlandi et al., 2013), more than half of TRPM1 is found in BPC bodies (Morgans et al., 2009; Koike et al., 2010b; Agosto et al., 2018; Fig. 1*A*). There have been some data suggesting that somatic TRPM1 is at least partially intracellular (Pearring et al., 2011; Xu et al., 2012), but the subcellular localization is unknown, as is the location of plasma membrane insertion. In this study, we have shown that the endoplasmic reticulum (ER) in ON-BPCs extends into axons and dendrites, and that TRPM1 in cell bodies is intracellular and colocalized with the ER. In addition, we have confirmed that the intracellular topology of ER-resident TRPM1 conforms to that of the superfamily of ion channels to which it belongs.

## Materials and Methods

### Animals

WT C57BL/6 mice were obtained from the Baylor College of Medicine Center for Comparative Medicine, and WT CD-1 mice were purchased from Charles River Laboratories (RRID: IMSR_CRL: 22). Nob3 mice (Grm6^nob3^; Maddox et al., 2008) were obtained from The Jackson Laboratory (RRID: IMSR_JAX: 016883). Absence of the Pde6b^rd1^ allele was confirmed by genotyping as previously described (Giménez and Montoliu, 2001), and absence of the Crb1^rd8^ allele (Mattapallil et al., 2012) was confirmed by PCR amplifying and sequencing the surrounding region of genomic DNA. All immunofluorescence microscopy experiments were performed with WT C57BL/6 mice aged 4–12 weeks, except experiments involving subretinal injections, for which CD-1 mice were used as described below. Mice of either sex were used for all experiments. All procedures were approved by the Baylor College of Medicine Animal Care and Use Committee.

### Cells

Human embryonic kidney (HEK293) cells were obtained from the ATCC (#CRL-1573, RRID: CVCL_0045) and maintained in DMEM (Corning) supplemented with 10% FBS (HyClone or Sigma). For fluorescence protease protection (FPP) assays, HEK293 cells were a gift from Michael Xi Zhu (The University of Texas Health Science Center at Houston, Houston, TX). Chinese hamster ovary (CHO) cells were maintained in Ham’s F12 Nutrient Mixture (HyClone), or DMEM/F12 50/50 (Corning), with 10% FBS.

### Primary antibodies

Antibodies, concentrations used, and validation information are shown in Table 1. Generation of monoclonal antibodies with full-length mouse mGluR6 has been described, and clone 312 was used previously (and validated) for Western blotting (Agosto et al., 2018). In this study, we used clone 366, which, unlike clone 312, performs well in retina immunostaining; clone 366 is validated in Figure 4. TRPM1, mGluR6, and 1D4 antibodies were purified from hybridoma cultures as previously described (Agosto et al., 2014).

**Table 1.** Primary antibody sources, concentrations used, and validation information

Antibody name (clone/designation)	Source/citation	Concentration	Validation
TRPM1 (545H5)Ms monoclonal	Agosto et al. (2014, 2018)	5 μg/ml, tissue 2 μg/ml, cells	WB and IF of Trpm1 KO mouse retina (Agosto et al., 2014, 2018)
mGluR6 (366)Ms monoclonal	Agosto et al. (2018); this study	5 μg/ml	IF of Grm6^nob3^ mouse retina (this study; Fig. 4)
Na^+^/K^+^ ATPase (H-300)Rb polyclonal	Santa Cruz Biotechnology #sc-28800, RRID:AB_2290063	4 μg/ml	Expected pattern of plasma membrane staining (Robillard et al., 2012; Han et al., 2013; Iwai-Takekoshi et al., 2016)
PKCαRb polyclonal	Cell Signaling #2056, RRID: AB_2284227	1:200	WB of macrophages from PKCα KO mouse (Koh et al., 2017); characteristic labeling of rod BPCs in mouse retina (Lu et al., 2013; Ribic et al., 2014; this study; Fig. 1)
1D4 tagMs monoclonal	Molday and MacKenzie (1983); Mackenzie et al. (1984); Hodges et al. (1988)	1 μg/ml	WB of rhodopsin KO mouse retina (Concepcion and Chen, 2010); specificity for recombinant tagged proteins (Wong et al., 2009; Agosto et al., 2014; Huynh et al., 2016)
HA tag (F-7)Ms monoclonal	Santa Cruz Biotechnology, #sc-7392, RRID: AB_627809	0.4 μg/ml	Specificity for recombinant tagged proteins (Zhao et al., 2017; Consalvi et al., 2017)
Ribeye A-domainRb polyclonal	Synaptic Systems, #192-103, RRID: AB_2086775	1 μg/ml	Correct size band on WB of retina tissue, compared to other antibodies detecting ribeye (Hubler et al., 2012); characteristic labeling of synaptic ribbon (Regus-Leidig et al., 2010; Anastassov et al., 2017; this study; Fig. 4)
BiP/GRP78Rb polyclonal	Abcam, #ab21685, RRID: AB_2119834	1 μg/ml	Colocalization with Sec61β in CHO cells and mouse retina, correct size band on WB of retina tissue (this study; Fig. 5)
GM130 (35/GM130) Ms monoclonal	BD Biosciences, #610822, RRID: AB_398141	2.5 μg/ml	WB and IF of GM130 KO mouse testes (Han et al., 2017); IF of GM130 KO liver cells (Park et al., 2018)
Gαo (2A)Ms monoclonal	Millipore, #MAB3073, RRID: AB_94671; Li et al. (1995)	5 μg/ml	Correct size band on WB of bovine and rat brain membranes, specificity for Gαo vs. other Gα subunits (Li et al., 1995); widely used marker for ON-BPCs (Dhingra et al., 2002; Huang et al., 2003; Xu et al., 2012; Neuillé et al., 2015); similar staining pattern as another antibody validated by IF of Gαo KO retina (Dhingra et al., 2000)
Gβ3 (C-16)Rb polyclonal	Santa Cruz Biotechnology, #sc-381, RRID: AB_2109618	5 μg/ml	WB of Gβ3 KO mouse retina (Dhingra et al., 2012); IF of Gβ3 knockdown myocytes (Macrez et al., 1999); specific staining of ON-BPCs and cone outer segments (this study; Fig. 8), similar to another antibody validated by IF of Gβ3 KO retina (Dhingra et al., 2012)
Gγ13Rb polyclonal	Novus, #NBP1-98572	5 μg/ml	Similar staining pattern as another antibody validated by IF of Gγ13 KO retina (Ramakrishnan et al., 2015)

Ms, mouse; Rb, rabbit; WB, western blotting; IF, immunofluorescence.

### Expression constructs

Cloning of TRPM1 (isoform C, NP_001034193.2) and NYX (NP_775591.1) from mouse retina was previously described (Agosto et al., 2014, 2018). DsRed cDNA was obtained from pDsRed-monomer-C1 (Clontech). Constructs for transfection of HEK and CHO cells were all cloned in pCDNA3.1(+) using KpnI and NotI, and consisted of free GFP or DsRed; untagged TRPM1; TRPM1 with a C-terminal 1D4 tag or N-terminal HA tag; EGFP-TRPM1 and TRPM1-EGFP fusions containing linkers GGSGG and APVAT, respectively; EGFP-NYX with EGFP behind the signal sequence (NYX[1-19]-EGFP-GGGSGGG-NYX[20-476]); NYX-EGFP (NYX-GGGSGGG-EGFP); or dsRed-NYX (Nyx[1-19]-DsRed-Nyx[20-476]). An Emerald-Sec61β construct with CMV promoter (mEmerald-Sec61-C-18) was obtained from Michael Davidson (Addgene plasmid #54249). pGrm6P-Emerald-Sec61β was constructed by cloning Emerald-Sec61β into a plasmid containing the *Grm6* promoter 200-bp critical region and SV40 enhancer (Kim et al., 2008), derived from Addgene plasmid #18817, a gift from Connie Cepko, by removing the GFP, IRES, and alkaline phosphatase portions of its sequence.

### Subretinal injection and electroporation

DNA for injection was prepared using a Qiafilter Maxiprep kit (QIAGEN) and dissolved in water. Injections and electroporations were performed as previously described (Matsuda and Cepko, 2004, 2008). Briefly, left eyelids of CD-1 P0 mouse pups were opened with an incision along the future edge of the eyelid. Pilot holes were made in the sclera (postlimbus) with a 30-G needle, followed by positioning of a 33-G blunt injection needle into the subretinal space. Approximately 450 nl of pGrm6P-Emerald-Sec61β plasmid DNA (2.5-3 mg/ml) in PBS with 0.1% Fast Green dye were injected using a microinjector (UMP3 Microsyringe Injector and Micro4 Controller, World Precision Instruments) set at 130 nl/s. Five 50-ms pulses of 80 V, separated by 950-ms intervals, were applied across the eyes using custom tweezers with 7-mm diameter electrodes and an ECM 830 square wave electroporator (BTX Harvard Apparatus). Eyecup dissections were performed approximately four weeks later.

### Retina immunostaining

Intact eyes from room-light-adapted animals were used. No obvious differences in TRPM1 localization were observed in light- and dark-adapted animals. Eyes were fixed in 4% PFA in PBS for ∼45–60 min, washed extensively in PBS, then cryoprotected in 30% sucrose in PBS overnight at 4°C. The cornea and lens were removed, and eyecups were embedded in OCT; 8- to 20-μm cryostat sections were adhered to Superfrost Plus slides (VWR or Fisher) or coverslips coated with 100 μg/ml poly-D-lysine (PDL). Sections were postfixed in 2–4% PFA for 10 min, washed in PBS, and blocked for 2 h at room temperature (RT) in PBS with 10% donkey serum, 5% BSA, and 0.2% Triton X-100. Samples were incubated overnight at 4°C with primary antibodies diluted in blocking buffer, washed in PBS, then incubated at RT for 2 h with secondary antibodies (donkey anti-mouse-Alexa Fluor 488 and goat or donkey anti-mouse-Alexa Fluor 555, or goat anti-mouse-IgG2B-Alexa Fluor 555 and goat anti-mouse-IgG1-Alexa Fluor 488; Invitrogen/Thermo Fisher) diluted to 8 μg/ml in blocking buffer, then washed in PBS and mounted with Prolong Gold (Invitrogen/Thermo Fisher).

For super-resolution stochastic optical reconstruction microscopy (STORM), #1.5 square coverslips were acid washed, then coated with 100 μg/ml PDL for 1 h, washed in water, and air dried. 8-μm cryostat sections were adhered to the coverslips, which were then glued with epoxy to the bottom (outside) of 35-mm hole-bottom dishes. Immunostaining was performed as described above, except that secondary antibodies were goat anti-mouse[F(ab’)2]-Alexa Fluor 647 and goat-anti-rabbit[F(ab’)2]-Alexa Fluor 555 (Invitrogen/Thermo Fisher). Before imaging, labeled sections were covered with imaging buffer [50 mM Tris (pH 8) 10 mM NaCl, 0.56 mg/ml glucose oxidase (Sigma), 34 μg/ml catalase (Roche or Sigma), 10% (w/v) glucose, 15 mM cysteamine hydrochloride, and 10% VECTASHIELD H-1000 (Vector Laboratories); Dempsey et al., 2011] and a second coverslip sealed with epoxy. The VECTASHIELD in the imaging buffer enables the photoswitching behavior of Alexa Fluor 555 (Olivier et al., 2013).

### Retina dissociation and immunostaining

Three mice were used for each experiment. Retinas were dissected in dissection/dissociation buffer [DD; 97.5% Hanks’ balanced salt solution, 0.11 mg/ml sodium pyruvate, 0.1% (w/v) glucose, 9.8 mM HEPES pH 7.3; Beaudoin et al., 2012] and washed three times in DD. Retinas were then suspended in 1 ml DMEM with 2 mg/ml freshly-added papain (Worthington) and incubated with the lid open in a 37°C 5% CO_2_ incubator for 30 min. The papain was inactivated by washing twice with 1 ml DMEM + 10% FBS. Cells in 1 ml fresh DMEM + 10% FBS were then dissociated by sharply tapping the tube ∼10 times on a hard surface (Berntson et al., 2003). Large debris were allowed to settle, and 150 μl of the supernatant were spread onto Superfrost Plus slides with a wide-orifice pipet tip, then allowed to adhere for 15 min at RT. 150 μl of 4% PFA in PBS was added directly to the cells, and incubated for an additional 15 min, then slides were washed in PBS and immunostained as described below for cultured cells.

### Cell transfection and immunostaining

Cells in 24-well plates were transfected with 0.1 μg mEmerald-Sec61-C-18 and 0.5-0.7 μg pCDNA3.1-TRPM1 plasmid DNA using Lipofectamine 2000 (Thermo Fisher) according to the manufacturer’s instructions. The next day, cells were briefly trypsinized and replated on PDL-coated (HEK cells) or uncoated (CHO cells) coverslips. At ∼48 h post-transfection, cells were fixed with 2% PFA in PBS for 10 min, washed in PBS, blocked/permeabilized with PBS + 1% BSA + 0.1% Triton X-100 (PBSAT) for 15 min, labeled with primary antibody in PBSAT for 45–60 min, followed by secondary antibodies 2–4 μg/ml in PBSAT for 30 min. For some experiments, CHO cells were transfected on coverslips, and labeled after ∼24 h without replating. Coverslips were mounted with Prolong Gold.

### Confocal microscopy and image processing

Images were acquired with a TCS-SP5 laser scanning confocal microscope (Leica) using a 63× oil immersion objective (Leica, HC PL APO CS2 63.0×, numerical aperture 1.40). Alexa Fluor 555 and Alexa Fluor 488 were detected in sequential mode with a 543 nm HeNe laser and 488 nm argon laser, respectively, with imaging parameters set to avoid cross-talk between the channels. Images were acquired with few or no saturated pixels. For figures, images were processed in ImageJ (NIH) and/or Photoshop (Adobe) to apply pseudocolors and adjust the minimum and maximum input levels, maintaining a linear slope. All images are single optical sections unless indicated as projections.

Line scans were determined from raw images in ImageJ using 1 px-wide lines and the “plot profile” tool. Cropped raw images of axons were straightened in ImageJ using 30 px-wide segmented lines and the “straighten” tool, and column average line scans were measured using the straightened axon image and plot profile. Profiles are shown normalized to the maximum intensity.

### STORM imaging and data analysis

Images were acquired with a Nikon N-STORM system, with a 100× oil immersion TIRF objective (CFI Apo TIRF, numerical aperture 1.49). 561 nm and 647 nm solid-state lasers were used at maximum power to induce photoswitching of Alexa Fluor 555 and Alexa Fluor 647, respectively, without the use of an activator fluorophore (Bock et al., 2007; Heilemann et al., 2008); 512 × 512 px fields (160 nm/px) were imaged with an Andor iXON DU 897 EMCCD camera and a quad cube filter (Chroma, zt405/488/561/640 m-TRF); 20,000–50,000 18-ms exposure frames (∼56 frames/s) were acquired for each channel, with alternating sequential activation. A cylindrical lens typically used for 3D-STORM was used, although data analysis was confined to 2D (see below). X-Y warp calibration was employed to correct chromatic aberration between channels. 2D-STORM reconstructions were performed with Nikon NIS Elements Ar Analysis software as previously described (Robichaux et al., 2017), except using minimum and maximum photon counts of 200–500 and 10,000, respectively. After reconstruction, density filters were applied to remove background events.

### Western blotting

Preparation of total retina lysate and Western blotting were as previously described (Agosto et al., 2018). Blots were incubated overnight with BiP antibody (Table 1), 1 μg/ml, then with horseradish-peroxidase conjugated anti-rabbit (Jackson ImmunoResearch), 0.16 μg/ml, followed by HyGLO (Denville Scientific) or SuperSignal West Pico (Thermo) chemiluminescent substrate, and exposed to film.

### FPP assay

HEK293 cells in 24-well plates were transfected with 0.4 μg EGFP-NYX or NYX-EGFP, or 0.6 μg EGFP-TRPM1 or TRPM1-EGFP, along with 0.2-0.4 μg dsRed; or 0.2 μg GFP and 0.4 μg dsRed-NYX; or 0.4 μg dsRed-NYX and 0.4 μg NYX-EGFP, using Lipofectamine 2000. The next day, cells were briefly trypsinized and replated in eight-well Lab-Tek II chambered coverglasses which had previously been coated with 100 μg/ml PDL. Assays were performed at RT, ∼48–52 h post-transfection. Immediately before imaging, wells were washed three times with RT KHM buffer (110 mM potassium acetate, 2 mM MgCl_2_, and 20 mM HEPES; pH 7.2) and left in 200 μl KHM for the assay. Digitonin (5% solution from Invitrogen/Thermo Fisher) was heated to 95°C to dissolve and diluted in KHM immediately before use. UltraClean Proteinase K (20 mg/ml, Mo Bio) was diluted in KHM immediately before use. Images were acquired with the TCS-SP5 laser scanning confocal microscope (Leica) using the 63× oil immersion objective described above, set to scan every 30 s. Following the 1-min scan, 200 μl of 200 μM digitonin was added to the well. Following the 2-min scan, 400 μl of KHM (no protease control) or 100 μg/ml proteinase K was added to the well. In most experiments, fields containing many cells (512 × 512 px, 481.5 nm/px) were imaged, and individual cells were boxed for data analysis. In some experiments, higher-magnification fields containing one to two cells (256 × 256 px, 120.6 nm/px) were imaged. In both cases, the duration of each scan was ∼10 s. Relative fluorescence *F* at each time point *t* was calculated as 100·(*F*_cell,t_ – *F*_bgd,t0_)/(*F*_cell,t0_ – *F*_bgd,t0_), where *F*_bgd,t0_ was determined from an area in the first frame containing no cells. Cells in which fluorescence of free dsRed or GFP did not decrease after addition of digitonin, indicating poor permeabilization, were omitted.

### Experimental design and statistical analysis

Microscopy images are representative examples of images from at least two independent experiments. For FPP assays, results from multiple wells from at least three independent experiments were combined.

## Results

### TRPM1 in ON-BPC bodies and axons is intracellular

To examine the location of TRPM1 in ON-BPC bodies, mouse retina sections were labeled with an antibody for TRPM1 that was previously validated with Trpm1 knock-out mice (Agosto et al., 2014, 2018), and antibodies for Na^+^/K^+^ ATPase, a marker for plasma membranes, or PKCα, a soluble cytoplasmic protein well characterized as a marker for rod BPCs. In cell bodies, there was little or no colocalization of TRPM1 with Na^+^/K^+^ ATPase, with TRPM1 appearing to be mostly intracellular (Fig. 1*B*). Labeling intensity was measured along lines drawn through the boundaries between cells; in most cases, the peak TRPM1 staining was noncoincident with the peak Na^+^/K^+^ ATPase staining (Fig. 1*B*). To more clearly resolve the TRPM1 location in cell bodies, we employed STORM (Rust et al., 2006; Dempsey et al., 2011). STORM reconstructions of retina sections labeled with TRPM1 and Na^+^/K^+^ ATPase antibodies clearly show non-overlapping staining in the cell bodies (Fig. 1*C*).

**Figure 2.**
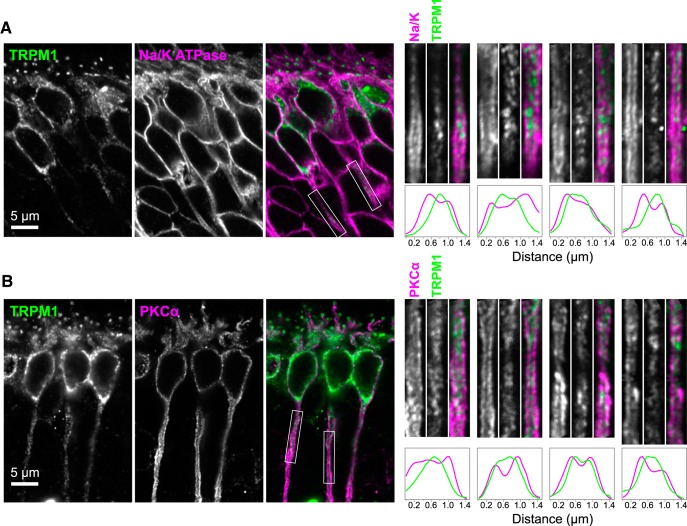
TRPM1 is intracellular in ON-BPC axons. Retina sections were labeled with TRPM1 (green) and antibodies to Na^+^/K^+^ ATPase to mark the plasma membrane (***A***) or PKCα to mark the cytoplasm (***B***; magenta). Regions containing axons were straightened, and normalized column average intensity profiles are shown. Two axons from the example images at left (regions indicated by boxes) as well as two axons from other images are shown.

Furthermore, if TRPM1 were localized to the plasma membrane, staining would be expected to be peripheral to PKCα. However, TRPM1 staining was mostly internal relative to PKCα in cell bodies (Fig. 1*D*). These results indicate that the majority of TRPM1 in cell bodies is located in intracellular membranes, and not in the plasma membrane. Column average intensity profiles of straightened axon segments show that TRPM1 labeling is mostly internal relative to both Na^+^/K^+^ ATPase (Fig. 2*A*) and PKCα (Fig. 2*B*) in axons as well.

### Heterologously expressed TRPM1 colocalizes with the ER

**Figure 3.**
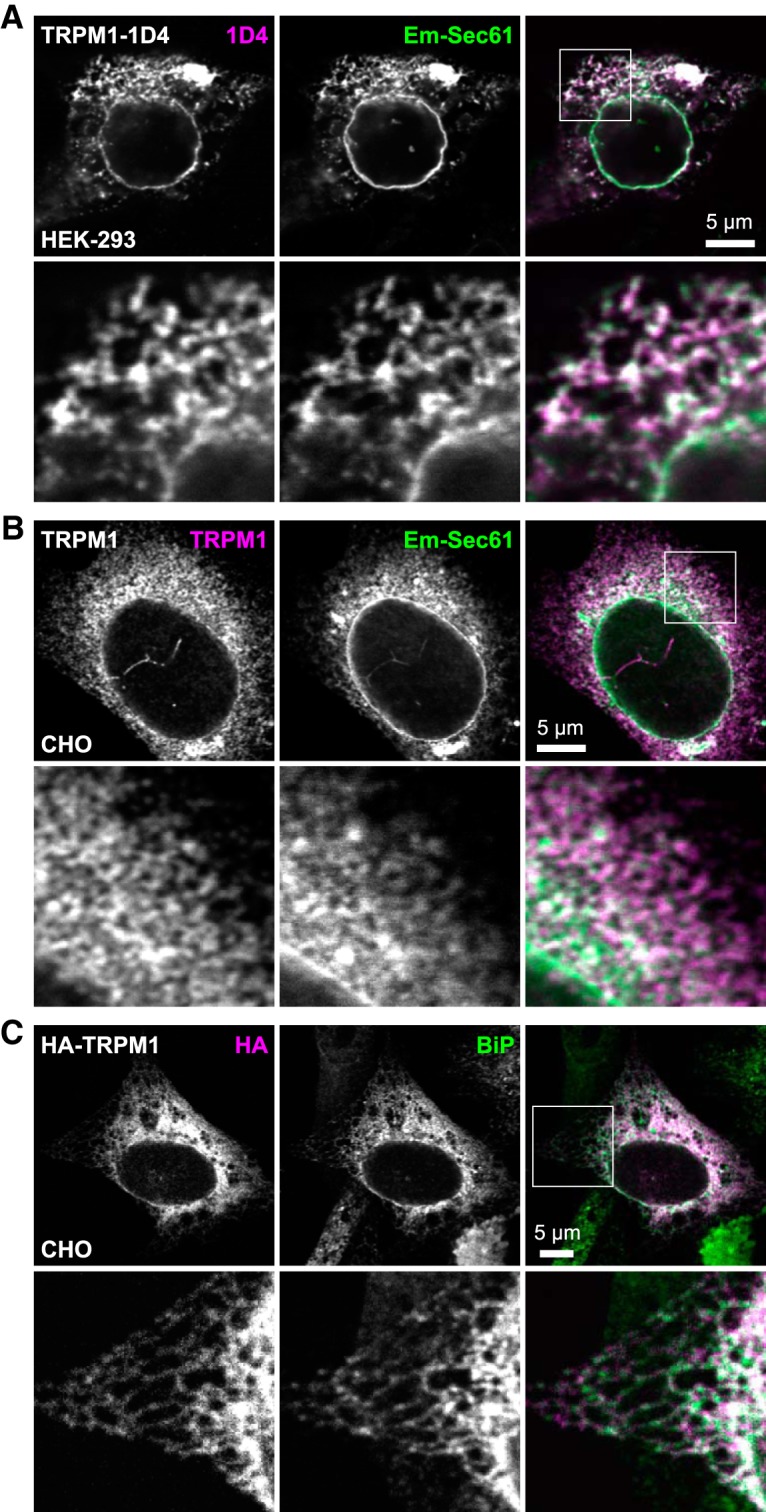
TRPM1 colocalizes with the ER in heterologous cells. ***A***, ***B***, HEK293 (***A***) or CHO (***B***) cells were transiently transfected with 1D4 epitope-tagged (***A***) or untagged (***B***) TRPM1 and Emerald-Sec61 (green). Cells were labeled with 1D4 (***A***) or TRPM1 antibody (***B***; magenta). ***C***, CHO cells were transiently transfected with HA-tagged TRPM1, and labeled with HA (magenta) and BiP (green) antibodies.

To determine the intracellular localization of TRPM1, we first examined the localization of heterologously-expressed protein. In both HEK293 and CHO cells transiently cotransfected with TRPM1 and Emerald-Sec61β, an ER membrane marker (Huang et al., 2016; Rapoport et al., 2017), extensive colocalization was observed throughout the ER network (Fig. 3*A*,*B*). TRPM1 staining also colocalized with antibody staining for endogenous binding immunoglobulin protein (BiP; Fig. 3*C*), which resides in the ER lumen and is involved in protein folding (Rapoport et al., 2017).

### ER in ON-BPCs extends into axons and dendrites, but not dendritic tips

**Figure 4.**
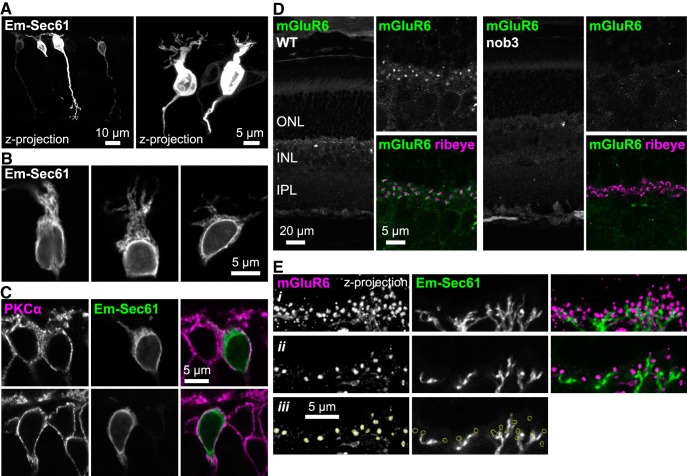
Localization of ER in ON-BPCs. Emerald-Sec61β was expressed in ON-BPCs by subretinal injection and electroporation of plasmid DNA using the ON-BPC-specific Grm6 promoter. ***A***, Z-projections of confocal stacks are shown oversaturated to highlight localization of Emerald-Sec61 in axons and dendrites. ***B***, Magnified views of cell bodies. ***C***, Immunostaining sections from injected retinas shows that in cell bodies, PKCα is located peripherally to the ER. ***D***, Validation of new mGluR6 mAb. Retina sections from WT (left) and nob3 (right) mice were labeled with mGluR6 antibody (green) and costained for presynaptic ribbon protein ribeye (magenta) to mark the location of synapses. Dendritic tip mGluR6 labeling is present in WT but not nob3 retinas. ***E***, Sections from injected retinas were immunostained with the mGluR6 mAb (magenta), revealing that Emerald-Sec61β in dendrites does not extend into the dendritic tip puncta. (*i*) Z-projection of confocal stack. (*ii*) Single optical slice from the same field. (*iii*) Same images as in (*ii*), with the location of mGluR6 puncta outlined in yellow.

**Figure 5.**
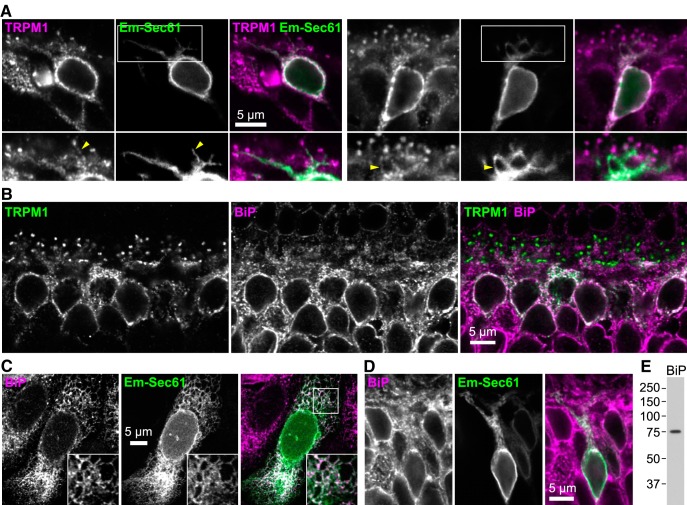
TRPM1 colocalizes with ER in ON-BPCs. ***A***, Sections from retinas expressing Emerald-Sec61β in ON-BPCs were immunostained with TRPM1 antibody (magenta). Boxes indicate regions of higher-magnification images (bottom). Colocalization is observed in cell bodies, and in some cases, dendrites (arrowheads). ***B***, Retina sections were immunostained with TRPM1 (green) and BiP (magenta) antibodies. ***C–E***, Validation of BiP antibody. ***C***, BiP immunostaining colocalizes with transfected Emerald-Sec61β in CHO cells. ***D***, BiP immunostaining colocalizes with Emerald-Sec61β in sections from injected retinas. ***E***, Western blotting of retina lysate (25 μg) probed with BiP antibody results in a band of the expected size (∼75 kDa; Munro and Pelham, 1986).

Little is known about the location of secretory pathway organelles, including the ER, in ON-BPCs. Immunostaining ER markers is challenging, because most cells have an extensive ER network and staining appears throughout the retina (Johnson et al., 2007; also see Fig. 5*B*), giving rise to extensive background signal from neighboring cells. Therefore, subretinal injection and electroporation of Emerald-Sec61β under control of the mGluR6 promoter (Matsuda and Cepko, 2004; Kim et al., 2008; Lagali et al., 2008; Van Wyk et al., 2015) were employed to specifically and sparsely label ON-BPCs with an ER marker (Fig. 4). Z-stack projections show Emerald-Sec61β localization throughout the cell bodies, dendrites, and axons of transduced cells (Fig. 4*A*). Even in cells with relatively weak expression of Emerald-Sec61β, signal is present in axons and dendrites (Fig. 4*A*, left), indicating the presence of ER in those compartments. Lacy structures characteristic of the ER network can be seen in higher-magnification images (Fig. 4*B*), and Emerald-Sec61β largely colocalized with the endogenous ER protein BiP (Fig. 5*D*). Labeling sections with PKCα antibody showed that as with TRPM1, Emerald-Sec61β in cell bodies is internal to PKCα (Fig. 4*C*).

To examine dendritic tips, we generated a monoclonal antibody against full-length mGluR6. This antibody labels dendritic tips as expected, and labeling is absent in Grm6*^nob3^* retina, which does not express mGluR6 protein (Maddox et al., 2008), demonstrating the specificity of the antibody (Fig. 4*D*). Labeling sections from injected retinas with mGluR6 antibody revealed no colocalization between Sec61β and mGluR6, indicating that the ER in dendrites does not extend into the dendritic tips (Fig. 4*E*). This is in contrast to PKCα, which can be seen clearly in the dendritic tip puncta (Fig. 1*D*, arrowheads).

### TRPM1 colocalizes with the ER in ON-BPC bodies

**Figure 6.**
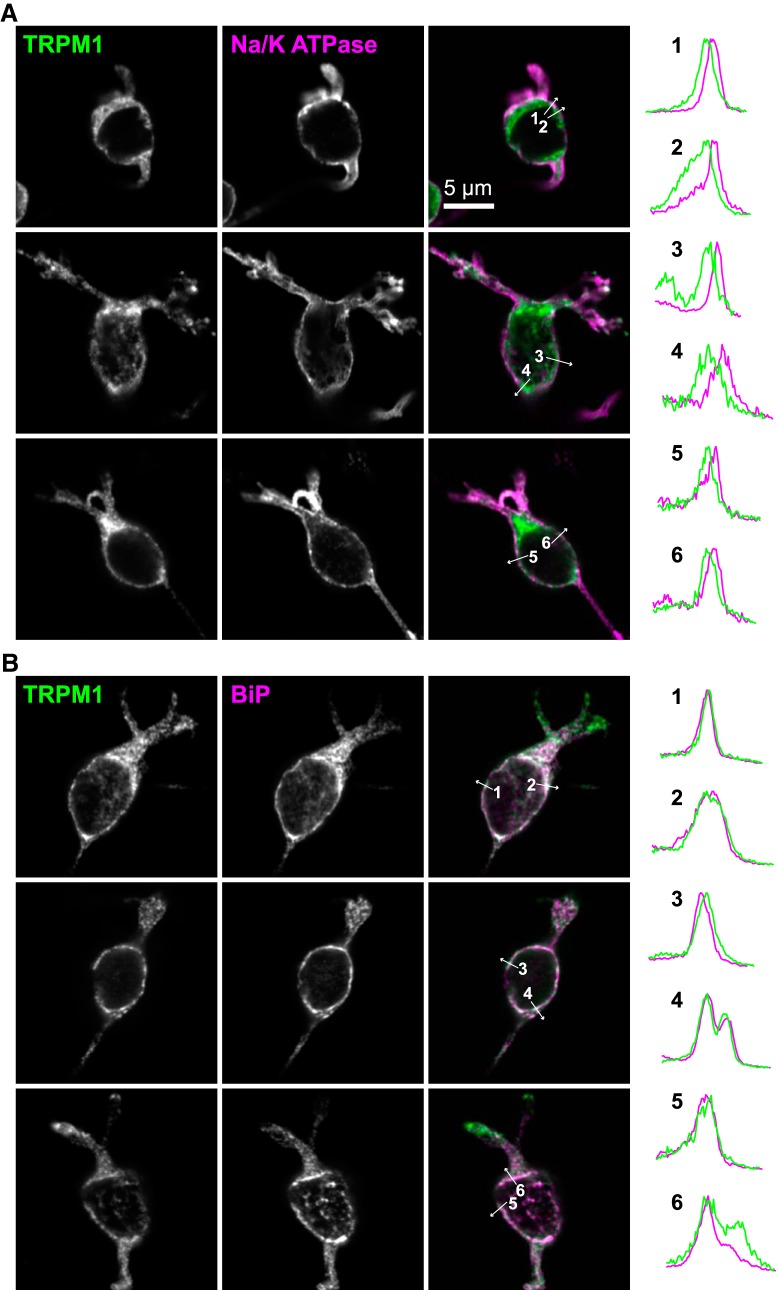
TRPM1 is intracellular and colocalized with ER in dissociated ON-BPCs. Acutely dissociated retinas were immunostained for TRPM1 (green) and Na^+^/K^+^ ATPase (***A***) or BiP (***B***; magenta). Intensity profiles were measured along lines indicated by the arrows on the merged image, and are shown normalized to the maximum height.

Sections from Emerald-Sec61β-injected retinas labeled with TRPM1 antibody show that in the cell bodies, TRPM1 largely colocalizes with the ER (Fig. 5*A*). In some cases, TRPM1 and Emerald-Sec61β also appear to colocalize in the dendritic shafts (Fig. 5*A*, arrowheads), although detection of TRPM1 in dendritic shafts is not reliable. To corroborate the ER localization of TRPM1, we costained retina sections with antibody for the ER lumen protein BiP (Fig. 5*B*). Validation of this antibody is shown in Figure 5*C–E*. The limited validation data for ER marker antibodies in retina tissue, as well as the fact that much of the ER labeling originates from other cell types not expressing TRPM1, makes the staining patterns difficult to interpret. However, in ON-BPC bodies there is at least partial colocalization of BiP with TRPM1, consistent with the the results from Emerald-Sec61β-injected retinas. There is no clear evidence of ER labeling in the ON-BPC dendritic tips, consistent with the absence of Emerald-Sec61β in the tips (Fig. 4*E*). Since interpreting immunostaining of ON-BPCs in retina sections may be complicated by the presence of other cell types, we also examined isolated ON-BPCs in acutely dissociated retina preparations (Fig. 6). Costaining with Na^+^/K^+^ ATPase confirmed largely intracellular somatic TRPM1 (Fig. 6*A*), which was partially colocalized with endogenous BiP (Fig. 6*B*), consistent with our previous observations.

### Golgi localization in ON-BPCs

**Figure 7.**
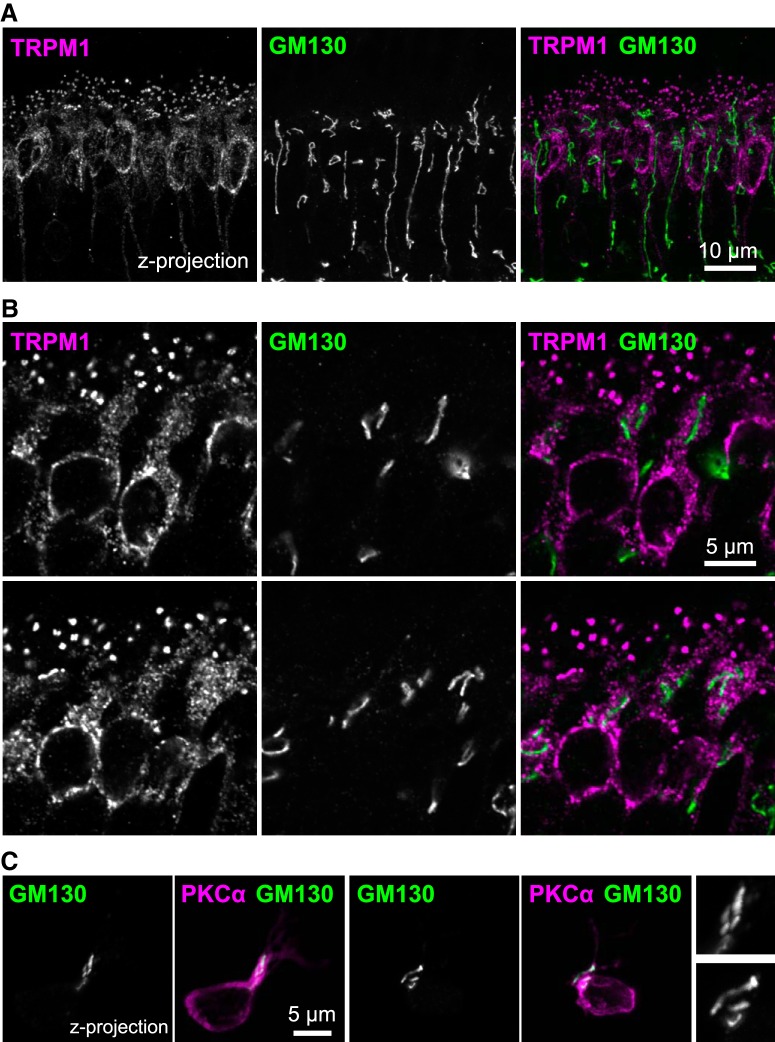
TRPM1 does not colocalize with Golgi in ON-BPCs. ***A***, ***B***, Retina sections were immunostained for TRPM1 (magenta) and cis-Golgi marker GM130 (green). ***C***, To confirm the localization of Golgi in ON-BPCs, acutely dissociated retina cells were immunostained for PKCα (magenta) and GM130 (green). Zoomed-in views of GM130-labeled structures are shown at right.

Immunostaining retina sections for the cis-Golgi marker GM130 (Nakamura et al., 1995) revealed filamentous or tubular structures throughout the inner nuclear layer, presumably present in various cell types (Fig. 7*A*); in ON-BPCs, GM130 appears to be detected mainly in the distal cell body and dendritic trunk region (Fig. 7*B*). The location of GM130 in ON-BPCs was confirmed in the absence of other cell types by immunostaining acutely dissociated retinal cells (Fig. 7*C*). There was no colocalization with TRPM1 (Fig. 7*B*).

### G protein subunits poorly colocalize with TRPM1

**Figure 8.**
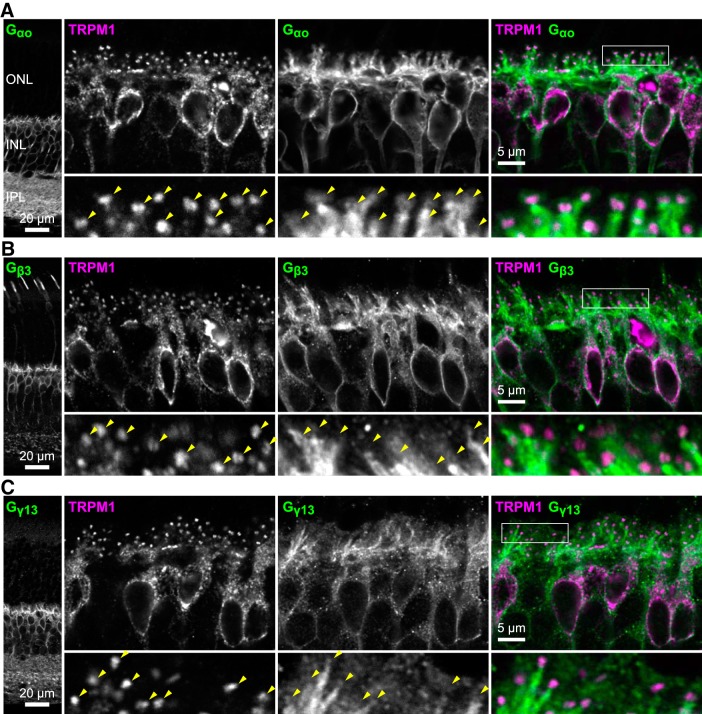
G protein subunits are poorly colocalized with TRPM1. Retina sections were immunostained for TRPM1 (magenta) and G_αo_ (***A***), G_β3_ (***B***), or G_γ13_ (***C***; green). Low-magnification images are shown at left, and boxed regions are shown in magnified views below. Arrowheads indicate dendritic tip puncta.

Heterotrimeric G protein subunits G_αo_, G_β3_, and G_γ13_ participate in the postsynaptic mGluR6 cascade in ON-BPCs (Dhingra et al., 2000, 2002, 2012; Huang et al., 2003; Ramakrishnan et al., 2015), and both G_α_ and G_βγ_ have been implicated in directly regulating TRPM1 (Koike et al., 2010a, 2010b; Shen et al., 2012; Xu et al., 2016). Although all three subunits are present in ON-BPC bodies and dendrites, there appears to be little colocalization with TRPM1 (Fig. 8) except in the dendritic tips, where G_αo_ is present (Fig. 8*A*, arrowheads). G_β3_ and G_γ13_ immunostaining was not sufficiently clear to confidently visualize them in the dendritic tips (Fig. 8*B*,*C*, arrowheads).

### TRPM1 N and C termini are cytoplasmic

**Figure 9.**
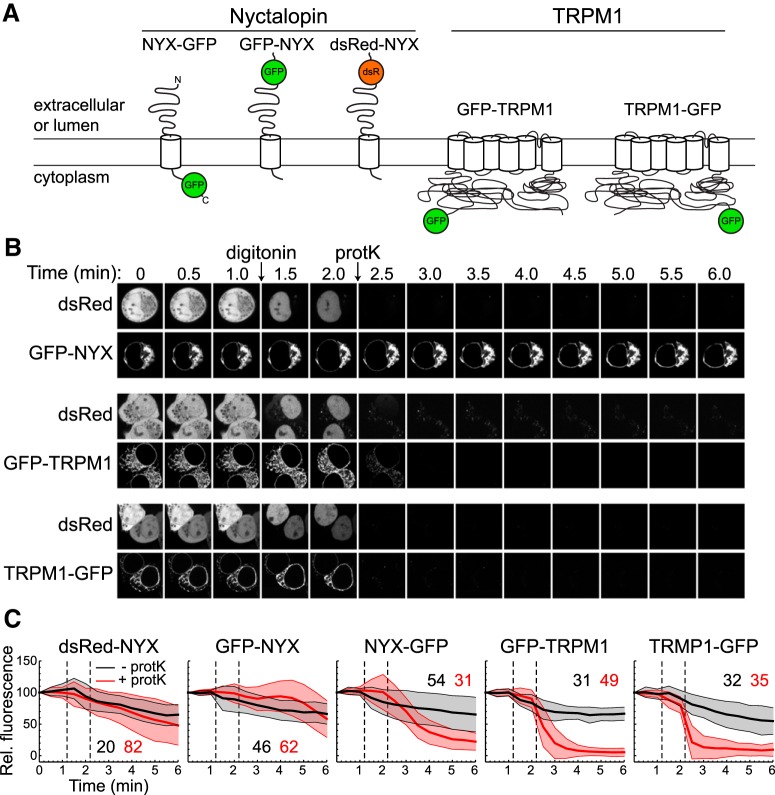
Topological analysis of heterologously expressed TRPM1. ***A***, Diagram of NYX and TRPM1 fluorescent fusions. ***B***, Example imaging timecourses of live cells. In some samples, cells were cotransfected with free dsRed to monitor digitonin action. On addition of digitonin to permeabilize the plasma membrane without disruption of the ER, dsRed diffuses rapidly out of the cytoplasm, then subsequently out of the nucleus. The fluorescence of GFP-NYX is resistant to proteinase K, implying GFP and the NYX N terminus are in the ER lumen, whereas that of GFP-TRPM1 and TRPM1-GFP is susceptible, revealing a cytoplasmic location for both C- and N-terminal GFP fusions. ***C***, Cells from image timecourses as in ***B***, as well as cells boxed from lower-magnification images, were combined, and fluorescence expressed as a percentage of the initial signal. Curves are the means of results from the number of cells indicated, with shaded regions representing mean ± 1 SD. Dashed lines following the 1- and 2-min time points indicate the approximate time of addition of digitonin and proteinase K, respectively.

Limited sequence similarity between the transmembrane (TM) domain of TRPM1 and those of other TRP family channels suggests that TRPM1 has six TM helices (Agosto et al., 2014; Guo et al., 2017). However, no experimental data regarding the topology of the protein have been reported, and different TM prediction algorithms yield varying results. A FPP assay (Lorenz et al., 2006; Tie et al., 2012; Nixon et al., 2013), in conjunction with live cell imaging, was used to assess the membrane topology of TRPM1 and NYX (Fig. 9). HEK293 cells expressing GFP fusion proteins were treated with the detergent digitonin to permeabilize the plasma membrane selectively without disrupting the ER, followed by proteinase K treatment to digest regions accessible to the cytoplasm. If the terminus fused to GFP is in the cytoplasm, it should be rapidly digested, measured as a decrease in fluorescence, whereas if the terminus is in the ER lumen, it should be protected from digestion. Mouse NYX, which is known to have an extracellular or ER-luminal N terminus and a cytoplasmic C terminus (Bojang and Gregg, 2012), was used as a control. As expected, the fluorescence of cells expressing NYX-GFP decreased rapidly on addition of protease, while that of cells expressing GFP-NYX or dsRed-NYX was resistant for several minutes. The fluorescence of cells expressing TRPM1 with GFP at either the N or the C terminus was sensitive to protease treatment, indicating cytoplasmic accessibility of both termini, consistent with an even number of transmembrane segments, almost certainly six, as found in TRP channels with known structures.

## Discussion

Our results demonstrate that TRPM1 in ON-BPC bodies is intracellular and located predominantly in the ER, and not the plasma membrane or Golgi apparatus (Figs. 1, 2, 5–7). This intracellular localization suggests that TRPM1 is inserted at the plasma membrane either in the dendrites or at the dendritic tips where it is concentrated. The subcellular localization of TRPM1 in dendritic shafts could not be determined, as TRPM1 signal is very poorly detected in dendrites, except at the dendritic tips. However, one hypothesis is that TRPM1 is trafficked all the way to the dendritic tips in the secretory pathway, where forward trafficking and/or plasma membrane insertion is triggered by a signal from a synapse-specific component. This hypothesis is consistent with the observations that knocking out other synaptic components reduces TRPM1 dendritic tip localization (Cao et al., 2011, 2015; Pearring et al., 2011; Xu et al., 2012; Gregg et al., 2014; Neuillé et al., 2015), if a correctly-formed synaptic complex is a prerequisite for TRPM1 plasma membrane insertion.

In other types of neurons, dendrites contain ER network, ER to Golgi intermediate compartments, and sometimes Golgi outposts. Synaptic proteins are trafficked by diverse secretory routes (for review, see Kennedy and Ehlers, 2006; Ramírez and Couve, 2011; Valenzuela and Perez, 2015) including long-haul transport or diffusion within dendritic ER (Cui-Wang et al., 2012; Valenzuela et al., 2014) and Golgi-independent secretory trafficking (Bowen et al., 2017). Secretory pathways in retinal BPCs are still relatively unexplored. We show that the exogenously-expressed ER protein Sec61β is present in somas, axons, and dendrites (Fig. 4), indicating that, as in other neurons, the ER network in retinal BPCs extends into axons and dendrites. The two ER markers employed, endogenous BiP and electroporated Sec61β, appear to have overlapping but nonidentical distributions, likely reflecting the heterogeneous nature of the ER. The widespread localization of TRPM1 in somas and axons (Figs. 1, 2) suggests that it may diffuse freely in a continuous ER network membrane. The identities of organelles participating in post-ER trafficking, and the signals that direct plasma membrane insertion, are unknown.

Also mysterious is the large ER pool of TRPM1. It may reflect merely an excess of trafficking or assembly intermediates; it would not be surprising to find biosynthetic intermediates of membrane proteins in the ER. On the other hand, the distribution of TRPM1 is notably different from other transmembrane participants in the synaptic signaling pathway, with more than half found in the cell bodies (Agosto et al., 2018). An alternative explanation is that TRPM1 may have a distinct function in the ER membrane. In melanocytes, functional roles for intracellular TRPM1 in melanin synthesis and calcium homeostasis have been reported (Oancea et al., 2009; Devi et al., 2009, 2013). The related TRPM channels TRPM2 (Lange et al., 2009; Manna et al., 2015) and TRPM8 (Bidaux et al., 2015) have also been shown to have intracellular functions. Like TRPM1, the heterotrimeric G protein subunits mediating synaptic transduction reside partially in cell bodies. However, no obvious colocalization was observed with somatic TRPM1 (Fig. 8), suggesting that if ER-resident TRPM1 does have a function, it may not depend on G_αo_, G_β3_, or G_γ13_.

TRPM1 is present in both rod and cone ON-BPC bodies (Fig. 1*D*, asterisks). Although the localization appears similar in all ON-BPCs costained with plasma membrane or ER markers, we did not specifically differentiate rod and cone BPCs. It is possible that these cells employ different routes and/or regulation of TRPM1 secretory trafficking or support different functions for intracellular TRPM1.

No trafficking signals have been identified yet in TRPM1. Cytoplasmic ER retention signals have been identified in numerous channel subunits including NMDA receptors (Standley et al., 2000; Scott et al., 2001, 2003), ATP-sensitive K^+^ channels (Zerangue et al., 1999), voltage-dependent Ca^2+^ channels (Bichet et al., 2000), voltage-gated Na^+^ channels (Zhang et al., 2008), and TRP channels (Köttgen et al., 2005). Topology analysis (Fig. 9) showed both N and C termini of TRPM1 are in the cytoplasm, as expected from structures of other TRP channels (Liao et al., 2013; Paulsen et al., 2015; Guo et al., 2017; Winkler et al., 2017; Autzen et al., 2018; Yin et al., 2018), and it is likely that one or both of the cytoplasmic arms contains sequences that direct and regulate forward trafficking. Deciphering sequence determinants of TRPM1 trafficking and its route to the dendritic tip plasma membrane will be important for understanding the maintenance of functional synapses in ON-BPCs.

## Synthesis

Reviewing Editor: Douglas Bayliss, University of Virginia School of Medicine Decisions are customarily a result of the Reviewing Editor and the peer reviewers coming together and discussing their recommendations until a consensus is reached. When revisions are invited, a fact-based synthesis statement explaining their decision and outlining what is needed to prepare a revision will be listed below. The following reviewer(s) agreed to reveal their identity: Joshua Singer. Note: If this manuscript was transferred from JNeurosci and a decision was made to accept the manuscript without peer review, a brief statement to this effect will instead be what is listed below.

This manuscript was seen by a new reviewer at eNeuro, who also appreciated the technical quality of the work and the insights provided regarding the pathway by which TRPM1 is trafficked to and inserted into postsynapses in ON bipolar cells. Some additional minor revisions were suggested to clarify the methods and results. These specific comments are provided below.

Reviewer #1

Advances the Field

The manuscript provides insight into the pathway by which TRPM1 is trafficked to and inserted into postsynapses in ON bipolar cells.

Comments to the Authors

Retinal slice experiments: were animals kept in the dark prior to dissection, or were they light-adapted? If the former, were dissections done under dim red or IR light? One might expect light-dependent differences in TRPM1 localization. Relatedly, could the results from double-labeling of G protein subunits (Figure 8) depend on ambient illumination?

Readers would benefit from some simple explanation-just a line or two will suffice-of each protein marker used: what it is/does, why it was chosen, etc... For example, in addition to BiP, PDI was used as a luminal marker for ER: why? Additionally, ribeye was used as a presynaptic marker but not mentioned in the results.

Aside from the mGluR6 experiments (Figure 4E), no controls for specificity were done in this paper (or I missed them). The topic of specificity in immunohistochemistry can trigger a lot of discussion and debate, but it is necessary, and the authors should address where the antibodies were validated.

A simple explanation of the FPP assay would be helpful.

The first subtitle in “Results” gives the impression that TRPM1 is only in axons and cell bodies, but it certainly is in dendrites. The subtitle should be edited.

A few edits to bring the issue of trafficking and insertion to the fore would be useful.
